# An efficient protein production system *via* gene amplification on a human artificial chromosome and the chromosome transfer to CHO cells

**DOI:** 10.1038/s41598-019-53116-2

**Published:** 2019-11-18

**Authors:** Takahito Ohira, Koichi Miyauchi, Narumi Uno, Noriaki Shimizu, Yasuhiro Kazuki, Mitsuo Oshimura, Hiroyuki Kugoh

**Affiliations:** 1 Department of Biomedical Science, Institute of Regenerative Medicine and Biofunction, Graduate School of Medical Science, Tottori University, Yonago, Tottori, 683-8503 Japan; 2 Chromosome Engineering Research Center, Tottori University, Yonago, Tottori, 683-8503 Japan; 3 Department of Applied Life Sciences, Tokyo University of Pharmacy and Life Sciences, Horinouchi, Hachioji, Tokyo, 192-0392 Japan; 4 Graduate School of Biosphere Science, Hiroshima University, Higashi-hiroshima, Hiroshima, 739-8521 Japan

**Keywords:** Genetic engineering, Biomaterials - cells, Expression systems

## Abstract

Gene amplification methods play a crucial role in establishment of cells that produce high levels of recombinant protein. However, the stability of such cell lines and the level of recombinant protein produced continue to be suboptimal. Here, we used a combination of a human artificial chromosome (HAC) vector and initiation region (IR)/matrix attachment region (MAR) gene amplification method to establish stable cells that produce high levels of recombinant protein. Amplification of Enhanced green fluorescent protein (EGFP) was induced on a HAC carrying *EGFP* gene and IR/MAR sequences (EGFP MAR-HAC) in CHO DG44 cells. The expression level of EGFP increased approximately 6-fold compared to the original HAC without IR/MAR sequences. Additionally, anti-vascular endothelial growth factor (*VEGF*) antibody on a HAC (VEGF MAR-HAC) was also amplified by utilization of this IR/MAR-HAC system, and anti-VEGF antibody levels were approximately 2-fold higher compared with levels in control cells without IR/MAR. Furthermore, the expression of anti-VEGF antibody with VEGF MAR-HAC in CHO-K1 cells increased 2.3-fold compared with that of CHO DG44 cells. Taken together, the IR/MAR-HAC system facilitated amplification of a gene of interest on the HAC vector, and could be used to establish a novel cell line that stably produced protein from mammalian cells.

## Introduction

Biotechnology and bioengineering enable artificial methods to modify the genetic material of living cells to produce novel compounds, and eventually contribute to development of recombinant protein production technology. Biopharmaceuticals such as recombinant proteins and nucleic acids are greatly expected to treat diseases with limited treatment options including some cancers^1^. In particular, monoclonal antibody (mAb) drugs, which have high effectiveness and few side effects, are commonly used in clinical trials and clinical practice^2^. However, unlike low-molecular weight medicines, the manufacturing process of bio-medicines is complicated and expensive^3^. Moreover, bio-medicines are produced by cells that may show instability of production of specific proteins during long-term culture^4^. Therefore, industrial preparation of mAbs requires production of cell lines that stably express high levels of protein using a technique that can be easily manipulated.

Chinese hamster ovary (CHO) cells are commonly used as a host cell for manufacturing of mAb drugs in the biopharmaceutical industry^5,6^. These cells have been used along with several methods that enhance protein expression^7^. A popular method is the use of methotrexate (MTX) to induce amplification of genes of interest in CHO DG44 cells^8^. CHO DG44 cells are deficient in dihydrofolate reductase (*dhfr*), and therefore, cells transfected with the gene of interest and *dhfr* can be selected in medium without hypoxanthine and thymidine, which are essential for nucleotide synthesis *via* the salvage pathway. Exposure to MTX, a high-affinity folate antagonist, can be used to amplify expression of a gene of interest around the *dhfr* locus^9^. However, the MTX gene amplification method takes a long time (at least 4 months)^10^. Therefore, establishment of isolated clones that stably produce high quantities of a protein of interest is considered a time-consuming and costly process.

In addition to the methionine sulfoximine (MSX), which Glutamine Synthetase (GS) inhibitor, gene amplification method is also widely used for recombinant antibody and protein production in mammalian cell culture. This system uses GS gene, which an enzyme produces glutamine from glutamic acid and ammonia. This synthesis pathway is essential for mammalian cells growth in glutamine lack condition. Thus, in containing MSX medium, mammalian cells depend on GS gene expression level for cell growth. MSX dose dependent exogenous GS gene amplification is induced with co-transfected an interest gene. MSX gene amplification method improved a time-consuming and costly process rather than MTX method^11^. However, it was reported that the production amount of the target protein decreased during culture for a long term from cells established by MST method. High producing subclones of recombinant CHO cells producing humanized antibody isolated at various MSX concentrations showed a significant decrease in production over the first six passages^12^.

Another gene amplification method uses a plasmid encoding a mammalian replication initiation region (IR) and a matrix attachment region (MAR), the sequence of which induces a spontaneous increase in the copy number of the gene of interest in animal cells (IR/MAR method). Initially, a IR/MAR sequence contained plasmid is maintained and multimerized at an extrachromosomal site and integrated into the host chromosome arm. In the latter context, the multimer initiates a breakage-fusion-bridge (BFB) cycle that generates chromosomal homogeneously staining regions, which are chromosome structures containing amplified genes^13^. This method of constructing homogeneously staining regions is simple, rapid, highly effective, and produces approximately 1,000 copies of transgenes within 1 month^14,15^. Accordingly, the IR/MAR gene amplification system has been used in basic cell biology research^13^, and has been adapted for recombinant protein production^14^. However, protein productivity and reactivity following gene amplification methods are different for different cell strains. For example, the IR/MAR sequence in CHO K1 cells induces weak gene amplification that is lower than that in CHO DG44 and COLO 320 cells^13–17^. On the other hand, production of recombinant proteins is higher in CHO K1 cells than in CHO DG44 cells^18^. Therefore, a cell line with sufficient protein productivity and gene amplification represents a powerful tool for production of recombinant proteins with the IR/MAR method.

General transfection of a constructed vector carrying a gene of interest into a host cell results in random integration into the host cell genome. However, because the majority of the genome consists of transcriptionally non-permissive heterochromatin, transgenes will likely be integrated into regions that are not favorable for high-level stable expression. Furthermore, even if the transgene is integrated into a transcriptionally active region, its expression status may still be silenced by a “position effect” involving epigenetic modification such as DNA methylation within the integrated transgene or promoter region^19–21^. Therefore, the expression profiles of transgenes differ depending on the chromosomal integration site. These positional effects result in variable expression levels in transfectant clones and the instability of recombinant protein productivity in long-term culture^22^. Thus, development of a new method that provides a stable supply of quantities of a desired protein of interest over the long term may contribute to additional development of mAb drugs.

Human artificial chromosomes (HAC), which are exogenous mini-chromosomes, are artificially created by chromosome engineering. HAC vectors have several advantages as gene delivery vectors, and they are stably and independently maintained in host chromosomes. The capacity to carry large genomic loci with their regulatory elements allows physiological regulation of the introduced gene in a manner similar to that of native chromosomes^23–25^. In addition, HAC vectors can be transferred into any cell line by microcell-mediated chromosome transfer (MMCT). We showed that the human factor FVIII (*FVIII*), also known as antihemophilic factor protein, from a HAC was expressed in a copy number-dependent manner in CHO K1 cells. When the HAC with *FVIII* (FVIII-HAC) was transferred from CHO K1 cells to human immortalized mesenchymal stem cells (hiMSC) using MMCT, *FVIII* was expressed at levels consistent with those of the original clones throughout 50 population doublings (PDL). Thus, the target gene on HAC, which is independent of the host chromosomes, could be maintained with long-term gene expression^26^. These results suggest that the HAC-mediated gene expression system may be a powerful tool for stable expression of transgenes, and possibly for industrial production of gene products.

A plasmid sequence containing EGFP gene was amplified with a combination of IR/MAR and a mouse artificial chromosome (MAC) using the CRISPR/Cas9 system. In this system, the efficiency of EGFP gene insertion in MAC vector were 40% and only EGFP signal intensity and fluorescence *in situ* hybridization (FISH) analysis were examined to assess gene amplification in this study^27^. Therefore, the potential for application to recombinant proteins remained unknown.

We report here that the combination of IR/MAR and HAC tools amplified the gene interest, and that the transfer of this HAC to CHO K1 cells has the potential to produce high levels of protein. In this study, we used Cre/loxP system, which high efficiency of site specific insertion system^28^, to insertion of interested genes in HAC vector. Additionally, we showed that this method was practicable to promote the production of proteins, not only EGFP but also recombinant secretory proteins such as mAbs. Thus, utilization of IR/MAR and an artificial chromosome may be an approach with significant potential for the development of therapeutic proteins.

## Results

### Establishment of a gene amplification method using the HAC vector

**Figure 1:**
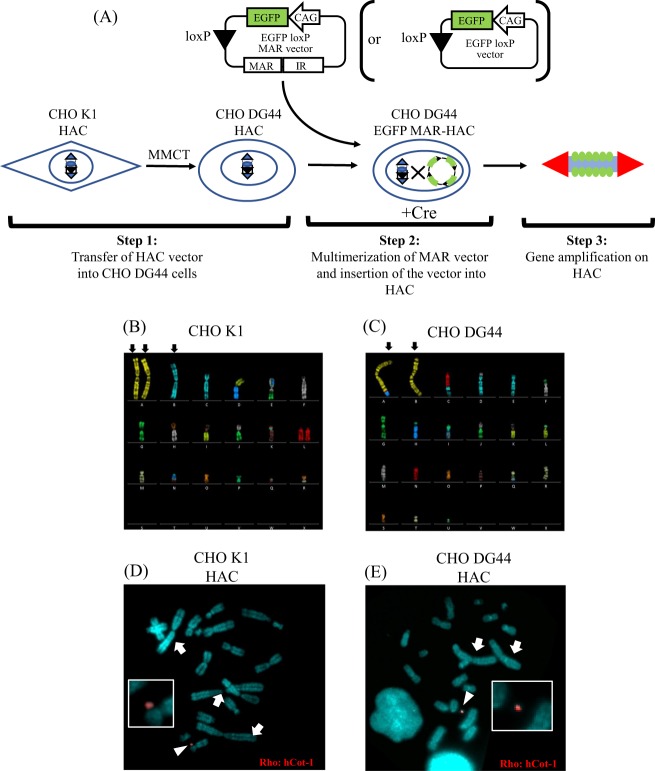
Schematic diagram of the IR/MAR HAC gene amplification system. (**A**) Step 1: HAC vector transfer from CHO K1 cells into CHO DG44 cells. Step 2: Multimerization of MAR vector and insertion of target genes into HAC. Step 3: Analysis of the effect of gene amplification on the gene expression level. The EGFP loxP MAR vector includes the CAG promoter to drive expression of *EGFP* cDNA, the IR/MAR sequence, and the loxP site. The EGFP loxP vector includes only the CAG promoter to drive *EGFP* cDNA and the loxP site as a control. Cre indicates use of the Cre recombinase for Cre-loxP site-specific recombination. (**B**) Multi-color FISH analysis in CHO K1 cells. The three large metacentric chromosomes (indicated by the arrows) characterize the karyotype of CHO K1 cells. (**C**) Multi-color FISH analysis in CHO DG44 cells. The two large metacentric chromosomes (indicated by the arrows) characterize the karyotype of CHO DG44 cells. (**D**) FISH analysis with a digoxigenin-labeled hCot-1 DNA (red) identified the HAC vector in CHO K1 cells (indicated by the arrowhead). The inset shows enlarged images of the HAC. The arrows indicate the three large metacentric chromosomes, which identify CHO K1 cells. (**E**) FISH analysis with a digoxigenin-labeled hCot-1 DNA (red) identified the HAC vector in CHO DG44 cells (indicated by the arrowhead). The inset shows enlarged images of the HAC. The arrows indicate two large metacentric chromosomes that identify CHO DG44 cells.

The strategy that we used to construct the HAC that carries the amplified target gene and IR/MAR is outlined in Fig. 1A. The efficiency of gene amplification by IR/MAR is dependent on the host cell. The effect of IR/MAR gene amplification is stronger in CHO DG44 cells compared with CHO K1 cells^13^. We first transferred the HAC vector from CHO K1 cells to CHO DG44 cells *via* MMCT (Fig. 1A: Step 1). We used multicolor FISH analysis and found significant differences between the karyotypes of CHO DG44 and CHO K1 cells. Three large metacentric chromosomes, which are easily distinguished from each other by size and centromere position, appeared in CHO K1 cell metaphase, whereas CHO DG44 cells had two chromosomes (Fig. 1B,C). To demonstrate that the HAC vector had been transferred from CHO K1 to CHO DG44 cells, we performed FISH analysis of microcell hybrid CHO DG44 clones. Retention of the HAC with the human Cot-1 (hCot-1) probe was detected independently of the host chromosomes both in microcell hybrid clones of CHO K1 cells with three large metacentric marker chromosomes and CHO DG44 cells with two marker chromosomes (Fig. 1D,E). The copy number of the HAC vector was uniformly one in more than 90% of tested metaphase spreads. These results demonstrated that the HAC vector was transferable from CHO K1 (Fig. 1D) to CHO DG44 cells (Fig. 1E) by MMCT.

### Amplification of *EGFP* on the HAC vector

**Figure 2:**
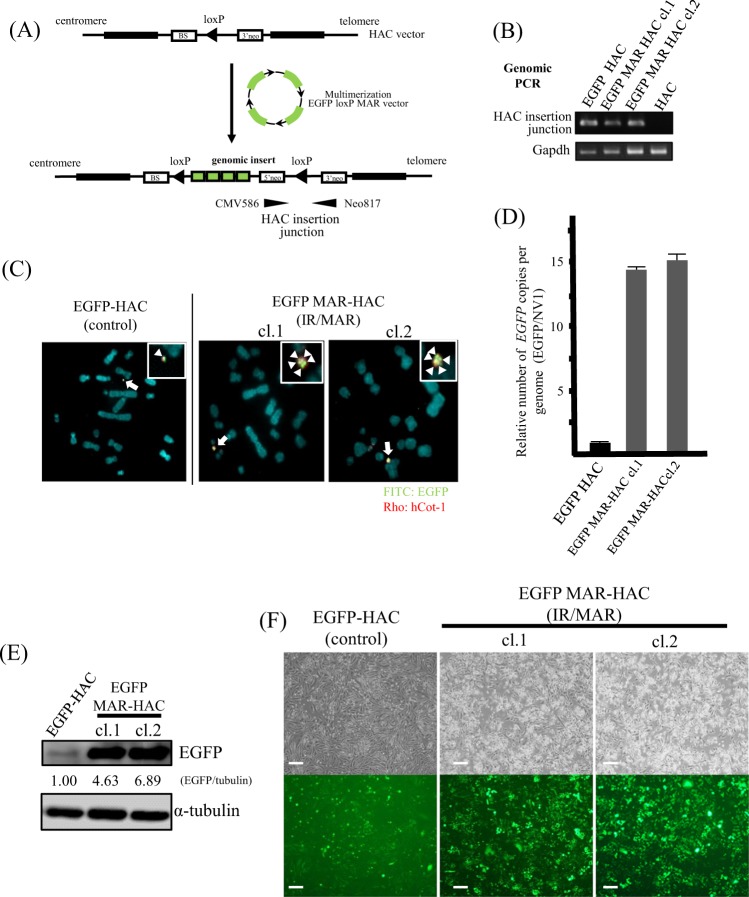
*EGFP* amplification on the HAC vector *via* the IR/MAR HAC system. (**A**) Schematic diagram of insertion of a plasmid encoding *EGFP* into the HAC vector. A circular targeting construct encoding *EGFP*, the non-functional neo gene, and the loxP site targeting the HAC vector are shown. Cre recombinase-catalyzed integration regenerates a functional neo gene. The resulting inserted allele is shown at the bottom. (**B**) The site-specific insertion event was detected in G418-resistant clones with genomic PCR analysis. A primer was designed to span the HAC insertion junction-specific site (indicated by a thin arrowhead in A). EGFP HAC and EGFP MAR-HAC clones indicate G418-resistant CHO DG44 clones. HAC: parental CHO DG44 cells including an empty HAC were used as a negative control. Cropped gels were used in this figure. Original full-length gels are presented in Supplementary Fig. S1. (**C**) Two-color FISH analysis of the HAC vector in G418-resistant CHO DG44 clones was performed with digoxigenin-labeled hCot-1 DNA (red) and biotin-labeled *EGFP* (green). The arrow indicates the HAC, and the inset shows enlarged images of the HAC (EGFP probe-specific green signals are indicated by the arrowheads). The left panel shows EGFP HAC control cells. The middle and right panels show EGFP MAR-HAC clones. (**D**) Relative copy number analysis of *EGFP* on the HAC vector with qPCR. Data were normalized to *NV1*. The copy number of *EGFP* in the EGFP HAC clone was arbitrarily at set as 1. Bars are the means ± SD of three independent experiments. (**E**) Expression levels of EGFP were analyzed with western blotting. An EGFP HAC clone was used as a control. Expression levels of EGFP were normalized to levels of tubulin. Expression level of protein in the EGFP HAC clone was arbitrarily assigned as 1. Cropped blots were used in this figure. Original full-length blots are presented in Supplementary Fig. S2. Data shown are representative of at least three independent experiments. (**F**) Expression of *EGFP* was tracked in living cells with fluorescence microscopy. A representative photo of cells is shown. Scale bars: 100 μm.

The HAC vector contains a 3′neo-loxP cloning site that can be used to insert a circular DNA fragment using the Cre-loxP system^23–25^. To insert the IR/MAR sequence and *EGFP* as a target gene into the HAC vector, we constructed a plasmid containing the IR/MAR sequence, *EGFP*, and the loxP site (EGFP loxP MAR plasmid: see Materials and methods for details). CHO DG44 cells were transfected with the HAC including the EGFP loxP MAR plasmid and the *Cre* expression vector pBS185 plasmid. We prepared transfectant clones with the EGFP loxP plasmid without the IR/MAR sequence and pBS185 plasmid as a control (Fig. 1A: Step 2). Clones with site-specific insertion of *EGFP* and the MAR sequence into the HAC vector were selected with G418. These G418-resistant clones were analyzed with PCR using HAC junction-specific primers, FISH analysis with hCot-1 and *EGFP*-specific probes, and western blotting to assess the expression level of EGFP. Genomic PCR with HAC junction-specific primers (Fig. 2A) was positive in EGFP HAC control cells and EGFP MAR HAC clones (cl.1 and cl.2). *Gapdh* was used as an internal control (Fig. 2B). To determine the effect of the IR/MAR sequence on gene amplification on the HAC vector, we performed FISH analysis using an *EGFP* genomic probe and qPCR analysis using *EGFP* genome-specific primers. The hybridization signal of *EGFP* on the HAC was detected as a single spot on more than 90% of metaphase spreads in CHO DG44 EGFP-HAC control cells with FISH analysis. On the other hand, the *EGFP* signal on the HAC in CHO DG44 EGFP MAR-HAC clones (cl.1 and cl.2) was detected as multiple *EGFP* signal spots due to amplification of *EGFP* by the IR/MAR method (Fig. 1A: Step 3 and Fig. 2C). Moreover, qPCR showed that the *EGFP* genomic region was increased approximately 15-fold in CHO DG44 EGFP MAR-HAC clones compared to CHO DG44 EGFP-HAC control cells (Fig. 2D). *NV1* was used as an internal control for the CHO genome^29^. Additionally, to investigate the effect of amplification of *EGFP* on protein production in CHO clones, we performed western blotting analysis using an EGFP-specific antibody. The expression level of EGFP was increased 4.6- to 6.9-fold in CHO DG44 EGFP MAR-HAC clones compared to CHO DG44 EGFP-HAC control cells (Fig. 2E). These expression levels of EGFP were correlated with the fluorescent intensity level of EGFP (Fig. 2F). The site-specific insertion of the IR/MAR sequence and the target gene on the HAC vector produced enhancement of protein expression that accompanied gene amplification. We called this gene amplification method using the HAC vector the “IR/MAR HAC system” (Fig. 1A).

### Transfer of the MAR-HAC vector to CHO K1 cells with MMCT to promote protein production

**Figure 3:**
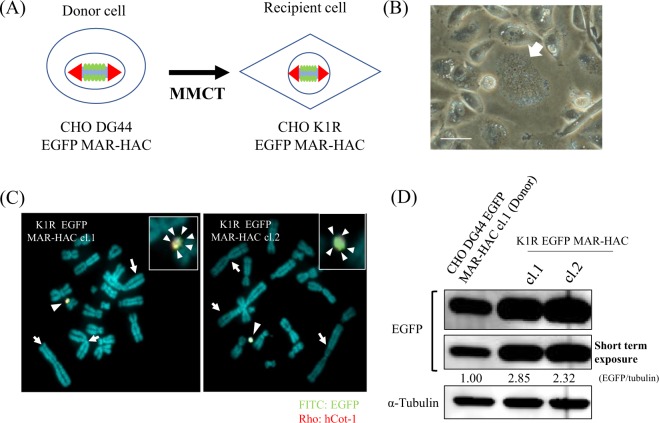
Transfer of the MAR HAC vector into CHO K1 cells promotes protein production. (**A**) Schematic diagram of transfer of the HAC vector in CHO DG44 cells to CHO K1 cells *via* MMCT. (**B**) Microcell formation was induced by colcemid treatment for 72 hr in CHO DG44 cells. Scale bars: 50 μm. (**C**) Two-color FISH analysis of the HAC vector in G418-resistant CHO K1 clones was performed with digoxigenin-labeled hCot-1 DNA (red) and biotin-labeled *EGFP* (green). The arrows indicate large metacentric chromosomes that identify the CHO strain. The left panel shows CHO K1R EGFP MAR-HAC cl.1. The right panel shows CHO K1R EGFP MAR-HAC cl.2. The arrowhead indicates the HAC, and the inset shows enlarged images of the HAC (EGFP probe-specific green signals are indicated by the small arrowhead). (**D**) Expression levels of EGFP were analyzed with western blotting. CHO DG44 MAR-HAC cl.1 (Donor cells) was used as a control. Expression levels of EGFP were normalized to levels of tubulin. The expression level of protein in DG44 EGFP MAR-HAC cl.1 was arbitrarily assigned as 1. Cropped blots were used in this figure. Original full-length blots are presented in Supplementary Fig. S3. Data shown are representative of at least three independent experiments.

The copy number of the gene and the location of integration into the host chromosome are factors that ultimately affect transgene expression in mammalian cells. In addition, the potential ability of the host cells to express the protein may also have a significant effect. In fact, the productivity of recombinant proteins is higher in CHO K1 cells than in CHO DG44 cells^18^. To investigate whether the recombinant protein level varies according to the cell strain, we transferred EGFP MAR-HAC from CHO DG44 to CHO K1 cells (Fig. 3A). It is very important for microcell formation ability of donor cells for chromosome transfer with MMCT to recipient cells. A high efficiency of microcell formation was observed using CHO DG44 cells as donor cells of the HAC vector (Fig. 3B). After MMCT, we established microcell hybrid CHO K1 clones (CHO K1R EGFP MAR-HAC cl.1 and cl.2) with *EFGP* and IR/MAR sequences using G418 selection. Multiple *EGFP* signals on HAC CHO K1R EGFP MAR-HAC cl.1 and cl.2 clones isolated from CHO DG44 cells were also observed (Fig. 3C). Additionally, EGFP levels were approximately 2.3- to 2.9-fold higher in CHO K1R EGFP MAR-HAC clones compared to the donor cell CHO DG44 EGFP MAR-HAC clone. EGFP signals in CHO K1R clones were too strong, therefore we also showed short-term exposure time data (Fig. 3D). These results suggested that the IR/MAR HAC system may enable large-scale production of recombinant protein by utilization of host cells with a high potential ability of protein expression.

### Stability of the MAR-HAC vector in CHO K1 cells

**Figure 4:**
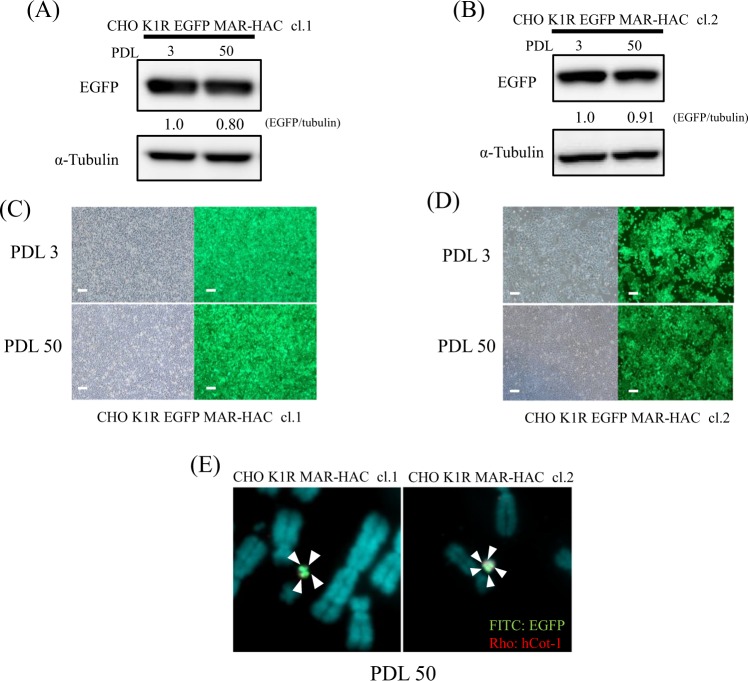
Analysis of EGFP expression during long-term culture of CHO K1R EGFP MAR-HAC clones. (**A**,**B**) Western blotting following in cell lysates from PDL 3 and 50 of long-term cultures. Expression level of EGFP was normalized to tubulin. Expression of protein at PDL 3 was arbitrarily assigned as 1. Panel (A) shows CHO K1R EGFP MAR-HAC cl.1. Panel (B) shows CHO K1R EGFP MAR-HAC cl.2. Cropped blots were used in this figure. Original full-length blots are presented in Supplementary Figs S4, S5. Data shown are representative of at least three independent experiments. (**C**,**D**) Expression of *EGFP* was tracked in living cells with fluorescence microscopy. A representative photo of cells is shown. Scale bars: 100 μm. Panel (C) shows CHO K1R EGFP MAR-HAC cl.1. Panel (D) shows CHO K1R EGFP MAR-HAC cl.2. (**E**) Mitotic stability of the EGFP MAR-HAC vector in CHO K1 cells. Two-color FISH analysis of the CHO K1R EGFP MAR-HAC clones at PDL 50 was performed with digoxigenin-labeled hCot-1 DNA (red) and biotin-labeled *EGFP* (green). EGFP probe-specific green signals are indicated by the small arrowheads. The left panel shows CHO K1R EGFP MAR-HAC cl.1. The right panel shows CHO K1R EGFP MAR-HAC cl.2.

To investigate that stability of gene expression by the MAR-HAC vector, we analyzed the protein production level and mitotic stability of the MAR-HAC vector using CHO K1 clones carrying EGFP MAR-HAC during long-term culture. EGFP expression was stably retained in CHO K1R EGFP MAR-HAC clones at 50 PDL (80%-91% expression compared with 3 PDL) (Fig. 4A,B). Furthermore, these expression levels of EGFP were correlated with fluorescent intensity levels (Fig. 4C,D). To investigate retention of the HAC vector during long-term culture, we performed FISH analysis using cells cultured for 50 PDL. The HAC was maintained with high frequency (90%) (Fig. 4E), suggesting results suggested that the protein production rate was stable from the MAR-HAC vector in the long-term culture.

### Anti-vascular endothelial growth factor (VEGF) antibody production using the IR/MAR HAC system

**Figure 5:**
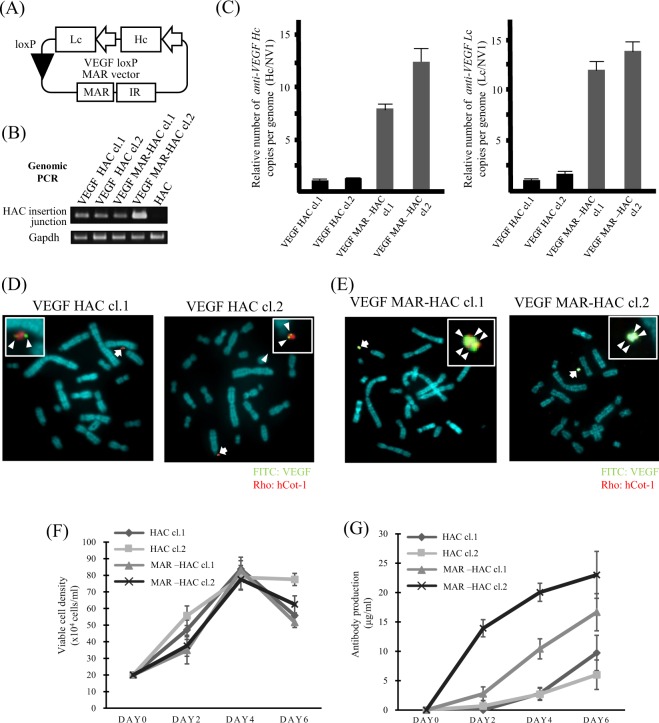
Anti-VEGF antibody production *via* the IR/MAR HAC system. (**A**) The VEGF loxP MAR vector includes the RPS7 promoter to drive anti-VEGF antibody *Hc* and *Lc* gene expression, the IR/MAR sequence, and the loxP site. (**B**) The site-specific insertion event was detected in G418-resistant clones with genomic PCR analysis. A primer was designed to span the HAC insertion junction-specific site. VEGF HAC clones and VEGF MAR HAC clones were G418-resistant CHO DG44 clones. HAC indicates the parental CHO DG44 cells including an empty HAC, which was used as a negative control. Cropped gels were used in this figure. Original full-length gels are presented in Supplementary Fig. S1. (**C**) Relative copy number analysis of anti-VEGF antibody *Hc* and *Lc* genes on the HAC vector with qPCR. Data were normalized to *NV1*. The copy number of anti-VEGF antibody genes in VEGF HAC clone 1 was arbitrarily at set as 1. Bars correspond to the means ± SD of three independent experiments. (**D,E**) Two-color FISH analysis of the HAC vector in G418-resistant CHO DG44 clones was performed with the digoxigenin-labeled hCot-1 DNA (red) and biotin-labeled anti-VEGF antibody gene (green). The arrow indicates the HAC, and the inset shows enlarged images of the HAC (anti-VEGF antibody probe-specific green signals are indicated by the arrowheads). Panel (D) shows VEGF HAC control clones. The left panel shows clone 1. The right panel shows clone 2. Panel (E) shows VEGF MAR-HAC clones. The left panel shows clone 1. The right panel shows clone 2. (**F**) Viable cell density in CHO DG44 VEGF HAC clones and CHO DG44 VEGF MAR-HAC clones. Bars correspond to the means ± SD of three independent experiments. (**G**) Analysis of anti-VEGF antibody production from CHO DG44 VEGF HAC clones and CHO DG44 VEGF MAR-HAC clones was measured using ELISA from day 2 to day 6. Bars correspond to the means ± SD of three independent experiments.

To further estimate the value of the IR/MAR HAC gene amplification system, we performed expression analysis using recombinant antibodies. We first constructed plasmid vectors that contain expression cassettes for the heavy chain (*Hc*) and light chain (*Lc*) genes encoding humanized anti-VEGF antibody, the loxP site, and the IR/MAR sequence (VEGF loxP MAR plasmid: see Materials and methods for details) (Fig. 5A). The anti-VEGF antibody *Hc* and *Lc* genome sequences were obtained from GeneBank (accession number: KX119517 and KX119516).

We generated CHO DG44 cells that carry the anti-VEGF antibody genes and the IR/MAR sequence on the HAC using co-transfection of the anti-VEGF antibody-encoding plasmid (VEGF loxP MAR or VEGF loxP) and the *Cre* expression vector. G418-resistant clones were analyzed using PCR with HAC junction-specific primers and FISH analysis. We established two CHO DG44 clones that had both anti-VEGF antibody genes and the IR/MAR sequence and two clones with only anti-VEGF genes on HAC. Genomic PCR with HAC junction-specific primers was positive in VEGF HAC cl.1 and cl.2, and VEGF MAR-HAC cl.1 and cl.2 (Fig. 5B). *Gapdh* was used as the internal control. Anti-VEGF antibody *Hc* and *Lc* genes were amplified to a greater extent in CHO DG44 VEGF MAR-HAC clones compared to CHO DG44 VEGF HAC control clones with qPCR analysis. The relative copy number of the anti-VEGF antibody *Hc* gene was increased 8.4-fold and 12.7-fold in VEGF MAR-HAC cl.1 and cl.2, respectively, compared to that in the VEGF HAC control (Fig. 5C). Likewise, the relative copy number of the anti-VEGF antibody *Lc* gene was increased 13.7-fold and 14.7-fold in VEGF MAR-HAC cl.1 and cl.2, respectively, compared to that in VEGF HAC cl.1 (Fig. 5C). HAC was present episomally, independent of the host chromosomes. Moreover, amplification of the anti-VEGF antibody genes confirmed that multiple anti-VEGF antibody gene signals for MAR-HAC were detected with FISH analysis (Fig. 5D,E).

To investigate the effect of gene amplification using the IR/MAR HAC system, we examined the growth properties of VEGF MAR-HAC and VEGF HAC control clones. No significant change was seen in the specific growth rate between VEGF-HAC and VEGF MAR-HAC clones (Fig. 5F). To further investigate whether production of anti-VEGF antibody increased due to gene amplification, we performed an enzyme-linked immunosorbent assay (ELISA) using the culture medium of each CHO DG44 VEGF clone. CHO DG44 with VEGF MAR-HAC clones produced more antibody compared with VEGF HAC clones. The production of antibody in VEGF MAR-HAC cl.1 and c1.2 was 16.7 μg/ml and 23.0 μg/ml, respectively, at day 6. On the other hand, the production of antibody in VEGF HAC control clones was 9.7 μg/ml and 6.0 μg/ml at day 6 (Fig. 5G). Taken together, these results indicate that the IR/MAR HAC system can be applied to recombinant antibody production.

### Transfer of the VEGF-HAC vector into CHO K1 cells

**Figure 6:**
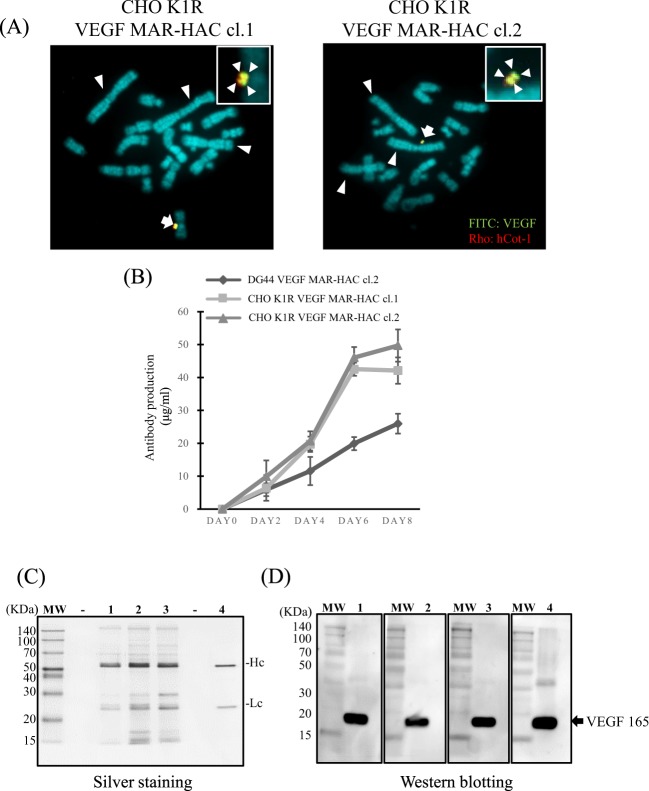
Transfer of the VEGF MAR-HAC vector to CHO K1 cells promotes antibody production. (**A**) Two-color FISH analysis of the VEGF MAR-HAC vector in G418-resistant CHO K1 clones was performed with digoxigenin-labeled hCot-1 DNA (red) and biotin-labeled anti-VEGF antibody gene (green). The arrow indicates the HAC, and the inset shows enlarged images of the HAC (anti-VEGF antibody probe-specific green signals are indicated by arrows). The arrowheads indicate the three large metacentric chromosomes that identify CHO K1 cells. The left panel shows CHO K1R VEGF MAR-HAC cl.1. The right panel shows CHO K1R VEGF MAR-HAC cl.2. (**B**) Analysis of anti-VEGF antibody production with ELISA from CHO K1R VEGF MAR-HAC clones and CHO DG44 VEGF MAR-HAC clones from day 2 to day 8. Bars correspond to the means ± SD of three independent experiments. (**C**) Culture supernatants were analyzed by SDS-PAGE. The protein bands were stained by silver staining. MW, molecular weight markers; line 1, antibody purified from CHO DG44 VEGF MAR-HAC cl.2; line 2, antibody purified from CHO K1R VEGF MAR-HAC cl.2; line 3, antibody purified from CHO K1R VEGF MAR-HAC cl.2; line 4, commercial human anti-VEGF antibody (bevacizumab; 0.1 µg). Hc means heavy chain. Lc means light chain. (**D**) Western blotting of the 19.1 kDa VEGF 165 protein with the purified antibody from the CHO cell clones. Line 1, antibody purified from CHO DG44 VEGF MAR-HAC cl.2; line 2, antibody purified from CHO K1R VEGF MAR-HAC cl.2; line 3, antibody purified from CHO K1R VEGF MAR-HAC cl.2; line 4, commercial human anti-VEGF antibody.

To investigate whether the mAb antibody production rate was promoted by chromosome transfer, we transferred VEGF MAR-HAC from CHO DG44 cells into CHO K1 cells. Using CHO DG44 VEGF MAR-HAC cl.2 as donor cells, we established two CHO K1 clones (CHO K1R VEGF MAR-HAC cl.1 and cl.2). FISH analysis indicated that the karyotype of the two clones had the characteristics of the CHO K1 strain and retained three large metacentric chromosomes. Additionally, multiple anti-VEGF antibody gene signals were detected on the HAC, and the HAC vector was independently retained from the host chromosomes in CHO K1 clones (Fig. 6A). We performed ELISA to measure anti-VEGF mAb produced by CHO KIR VEGF MAR-HAC clones. CHO K1 VEGF MAR-HAC clones produced more anti-VEGF antibodies compared with the donor clone (CHO DG44 VEGF MAR-HAC cl.2). The production of antibody by the K1R VEGF MAR-HAC cl.1 and c1.2 clones was 42.5 μg/ml and 46.1 μg/ml, respectively, at day 6. On the other hand, production of antibody by the donor clone (CHO DG44 VEGF MAR-HAC cl.2) was 19.9 μg/ml at day 6 (Fig. 6B). Thus, CHO K1 cells produced approximately 2.3-fold more VEGF mAb than CHO DG44 cells. These results suggested that the MAR-HAC vector including amplified target genes such as recombinant mAbs could be transferred to other mammalian cells, which will enable users to select cell lines to optimize production of recombinant proteins.

### The quality of the antibody produced by the IR/MAR HAC system

To investigate the quality of the anti-VEGF antibody produced by CHO DG44 and CHO K1R MAR-HAC clones, antibodies were purified from the culture medium of each clone using Protein G magnet beads. The SDS-PAGE analysis performed using the product on day 6 (Fig. 6C). The two major bands representing the Hc and Lc were detected in CHO DG44 VEGF MAR-HAC cl.2 (line 1) and CHO K1R VEGF MAR-HAC cl.1 and cl.2 (line 2 and line 3). These major bands were consistent with the commercial anti-VEGF antibody (bevacizumab). Furthermore, CHO K1R clones were detected to strong signal compared with CHO DG44 clone. This result correlated with the result of ELISA (Fig. 6B). Next, we examined the reactivity of the purified antibodies to VEGF antigen. As shown in Fig. 6D, the antibodies from CHO DG44 and CHO K1R clones recognized the 19.1-kDa VEGF 165 protein (line 1–3), as well as in the production of the commercial human anti-VEGF antibody (line 4). These results suggested that the antibody produced by IR/MAR HAC system was satisfactory in integrity and reactivity.

## Discussion

Recently, the great demand for therapeutic proteins has significantly advanced the manufacturing platform for recombinant protein production using the CHO cell culture system^6^. Several efforts, including cell engineering, optimization of cell line development, and cell culture medium optimization, have resulted in significant improvements in recombinant protein production^30–32^. However, recombinant cell lines developed for therapeutic antibody production often suffer from instability or lose protein expression during long-term culture^4^. In this study, we established CHO cell lines with efficient amplification of target genes that show stable levels of recombinant protein expression by combining the HAC and IR/MAR method. Furthermore, we showed HAC including amplificated interest genes could transfer to more appropriate cells for protein production using MMCT. These findings suggest that the IR/MAR HAC system has the potential to promote production of protein for industry needs.

The protein expression level in CHO DG44 cells with the IR/MAR HAC system, which was used to amplify *EGFP*, resulted in 4.6- and 6.9-fold increases compared to the control (Fig. 2A). In contrast, the protein expression level of anti-VEGF antibody expression using this system was approximately 2.0-fold higher compared to the control (Fig. 5G). The difference in protein expression levels may be attributed to protein profiles. A typical antibody protein is composed of two different gene products, the Hc and Lc chains. In this study, the gene amplification status of the anti-VEGF antibody genes was similar between *Hc* and *Lc* in the VEGF HAC clones (Fig. 5C). The balance of *Hc* and *Lc* gene expression is likely important for antibody protein production^33^. Accordingly, we cannot rule out the possibility that recombinant proteins such as antibodies may not be folded properly after expression process. Efficient expression of Hc and Lc requires an appropriate signal peptide for transport of Hc and Lc polypeptides to the endoplasmic reticulum for proper folding, assembly, and post-translational modification. Translocation of the nascent protein from the cytosol to the endoplasmic reticulum is mediated by the signal peptide and is an important step in protein secretion^34^. Therefore, selection of the best signal peptides for the IR/MAR HAC system may improve the production of secreted antibodies.

Optimization of the medium used for protein production is an important approach for increasing the protein concentration of a target recombinant protein^35^. Histone deacetylase inhibitors and retinoic acid enhance antibody production in CHO cells^36–38^. Additionally, the removal of animal-derived supplements was recommended because of safety concerns. Recently, many biopharmaceutical companies have shifted to the development of chemically defined medium formulations for antibody production in CHO cells^32^. Several serum-free and chemically defined CHO-specific cell culture media, as well as several medium systems combining basal media and feed, have become available. Moreover, the majority of the biopharmaceutical industry is currently using fed-batch suspension culture of recombinant CHO cells as a platform technology for biopharmaceutical protein production^39^. Although we used adherent CHO cells (CHO K1 and CHO DG44) in medium containing serum for establishment of recombinant protein produced with the IR/MAR HAC system, most industrial production of recombinant pharmaceuticals currently employs high-density suspension culture in serum-free medium as described above. Therefore, the combination of the IR/MAR HAC system and culture conditions including suspension culture, fed-batch, and optimized chemically defined CHO-specific cell culture media will improve the quality and productivity of protein derived from recombinant CHO or other mammalian cell lines.

It has been reported that recombinant protein production facilitated by improved the MTX method using CHO cell line^30,31^. A plasmid engineered vector was developed to the increased production of anti-VEGF antibody form CHO dhfr defect cell lines^30^. The engineered vector transfected CHO cells showed more highly sensitive to MTX and increased gene amplification. Anti-VEGF antibody was increased 1.6-fold using this engineered vector compared to that standard vector. The maximum production of recombinant protein (anti-VEGF antibody) was 35 μg/ml/day. In addition, cell system was developed that obtain high produces of targeted recombinant protein (anti-colon cancer antibody) by combination of Cre-loxP system and MTX method^31^. In this report, most productive clone was produced 400 μg/ml/3day. On the other hand, the maximum production of recombinant protein (anti-VEGF antibody) was 50 μg/ml/8day in this study using IR/MAR-HAC system (Fig. 6B). Thus, although IR/MAR-HAC system has not remarkable improved recombinant protein production rate compared to these previous studies^31^, MTX treatment is still time consuming. The MTX gene amplification method usually takes at least 4 months^10^. In IR/MAR HAC system, it reproducibly took 3–4 weeks to isolate a gene amplificated and recombinant protein produced CHO-DG44 cell line, and 3–4 weeks took to obtain MAR-HAC transfer to CHO-K1 cell line by MMCT (2 months in total). Additionally, those previous studies^30,31^ were not been shown to assess stability over long-term protein expression. We showed the protein production rate was stable from the MAR-HAC vector in the long-term culture (Fig. 4). Therefore, IR/MAR HAC system will benefit as the method that stably produce target proteins.

In this study, we used plasmid containing IR from DHFR for construction of the IR/MAR-HAC. Recently, G5 that lie in the beta-globin locus was identified as a core IR that favors gene expression like an enhancer. A long array of G5 provides an excellent environment for gene expression^40^. The sequences play a role of providing an artificial environment that promotes gene expression and avoids gene silencing. The expression level of a target gene (EGFP) from IR(G5)/MAR plasmid was even slightly higher than that from the IR(DHFR)/MAR plasmid^40^. Therefore, using the HAC vector including in IR(G5)/MAR-HAC system has a potential to promote production of recombinant protein and more stably expression in long-term culture. Thus, further studies that including improvement of IR/MAR-HAC system may provide a novel technology used for high produced recombinant protein mammalian cells.

## Materials and Methods

### Cell culture

CHO K1 cells were obtained from ATCC (Manassas, VA, USA), and CHO DG44 cells were obtained from Dr. Lawrence Chasin of Columbia University and Dr. Motonobu Katoh of our laboratory. These cells were maintained in Ham’s F-12 nutrient mixture (Invitrogen, Carlsbad, CA, USA) plus 10% fetal bovine serum (FBS) (JRH Biosciences, Lenexa, KS, USA). CHO K1 hybrids carrying the HAC (21HAC4) were cultured with 8 μg/ml blasticidin S hydrochloride (Wako, Tokyo, Japan) as previously described^24^. In antibody purification analysis, CHO K1 and CHO DG44 cells were maintained in Ham’s F-12 nutrient mixture (Invitrogen) 10% plus IgG free fetal bovine serum (FBS) (GE Healthcare, Piscataway, NJ, USA).

### Plasmid vector

The EGFP loxP vector was constructed as follows: loxP was amplified with PCR using the pPAC4 vector as a template with primers (5′-GAAGGCCTGGAGTTCCGCGTTACATAACTTACGGT-3′ and 5′-GAAGGCCTGGTGACCTAAGCTTGCATGCAACTTCGT-3′), digested with StuI (NEB, Hertfordshire, UK), and cloned into the StuI-digested pCAG-EGFP vector (pCAG-EGFP CMV loxP vector)^23,41^. The pPAC4 vector was obtained from the BAC PAC Resources Center at the Children’s Hospital Oakland Research^24^. To convert the ampicillin resistance gene (*Amp*) of the pCAG-EGFP CMV loxP vector into the kanamycin resistance gene (*Km*), *Km* was amplified with PCR using the pPAC4 vector as a template with primers (5′-AAAAGTACTCCAATTAACCAATTCTGATTAGAAAAACT-3′ and 5′-GGACTAGTAAAGCCACGTTGTGTCTCAAAATCTCTGAT-3′), digested with ScaI and SpeI, and cloned into the ScaI- and SpeI-digested pCAG-EGFP vector (EGFP loxP vector). The EGFP loxP MAR vector was constructed as follows: The EGFP loxP vector was digested with PstI and cloned into the PstI-digested pΔBN AR1-Dhfr vector^14^.

The VEGF loxP MAR vector was constructed as follows: a fragment obtained by digesting the inspB4ins2 vector with AvrII and SpeI was self-ligated to create a subcloning vector pB4ins1^41^. The A7 enhancer sequence fragment was amplified with PCR using the IMR90 cell genome as a template with primers (5′-ATTAATTAATCTTAGTATGGTAAACCTTTTGAAGTAGATTC-3′ and 5′-GTAACGCGTCAAGTTTTTATTTTGTTCTCACAATTAAGTCTATAC-3′), digested with MluI, and cloned into the NruI- and MluI-digested pB4ins vector (pB4-A7 vector)^41^. The cloning vector including the human RPS7 gene promoter and the anti-VEGF antibody *Hc* gene was prepared with a synthetic gene (pUCFa-RPS7-VEGF-Hc). The *RPS7* promoter sequence information was obtained from Gene Bank (Accession Number: NG_011744.1). The anti-VEGF antibody *Hc* gene sequence information was obtained from Gene Bank (Accession Number: KX119517). The cloning vector including the human *RPS7* gene promoter and anti-VEGF antibody *Lc* gene was prepared with a synthetic gene (pUCFa-RPS7-VEGF-Lc). The anti-VEGF antibody *Lc* gene sequence information was obtained from GeneBank (Accession Number: KX119516). The synthetic Lc vector pUCFa-RPS7-VEGF-Lc was digested with AscI and XhoI, and the synthetic Hc vector pUCFa-RPS7-VEGF-Hc was digested with MluI and XhoI. Then, each fragment was ligated to construct the pUC-Fa-RPS7-VEGF-LcHc vector. pB4-A7 was digested with NaeI and MluI, and pUC-Fa-RPS7-VEGF-LcHc was digested with MluI and SnaBI. Then, each fragment was ligated to construct the pUC-Fa-A7-RPS7-VEGF-LcHc vector. The AscI enzyme cut site was inserted into the PstI site in the pΔBN AR1-Dhfr vector, which was digested with AscI, and cloned into the AscI-digested pUC-Fa-A7-RPS7-VEGF-LcHc vector (VEGF loxP MAR vector). The VEGF loxP vector was constructed as follows: the VEGF loxP MAR vector was digested with HpaI and FseI, the fragment was blunted, and the blunted DNA was self-ligated.

### MMCT

MMCT was performed as previously described^23^. Briefly, the donor cells were incubated with 0.05 μg/ml colcemid (Sigma, St. Louis, MO, USA) in F12 (Invitrogen) containing 20% FBS (JRH Biosciences) for 72 hr. Micronuclei were harvested by treatment with 10 μg/ml cytochalasin B (Sigma) and centrifugation, and were sequentially filtered through 8, 5, and 3 μm polycarbonate filters (Whatman Nuclepore, Kent, UK). Fusion was mediated by 47% polyethylene-glycol 1000 (Wako), followed by extensive washing with serum-free DMEM (Sigma). After incubation for 24 hr in F12 supplemented with 10% FBS, cells with chromosome transfer were selected with G418 (Sigma). CHO DG44 cells were cultured with 300 μg/ml G418. CHO K1 cells were cultured with 800 μg/ml G418.

### Transfection

The CHO hybrids (4 × 10^5^ cells) containing the HAC were transfected with the EGFP loxP MAR (EGFP loxP, VEGF loxP MAR, VEGF loxP) vector and the Cre expression vector pBS185 CMV-Cre by lipofection with 2 µg EGFP loxP MAR (EGFP loxP, VEGF loxP MAR, VEGF loxP) plasmid and 1 µg pBS185 plasmid with 7.5 µl Lipofectamine 2000 reagent (Invitrogen) according to the manufacturer’s instructions^23^. After 24 hr in basic growth medium, cells were cultured in medium containing G418. Fourteen days later, drug-resistant colonies were picked and expanded for further analyses described below.

### FISH

FISH analyses were performed in fixed metaphases of microcell hybrids using digoxigenin-labeled (Roche, Basel, Switzerland) human Cot-1 DNA (Life Technologies, Carlsbad, CA, USA) and biotin-labeled EGFP loxP or VEGF loxP vector. The preparation of chromosomes, probe, hybridization, washing, and signal detection were performed according to our previous report^22^. Briefly, chromosomal DNA was counterstained with DAPI (Sigma). Images were captured using an AxioImagerZ2 fluorescence microscope (Carl Zeiss GmbH, Jena, Germany) and analyzed with the Ikaros software program (MetaSystems, Altlussheim, Germany). Multi-color FISH (mFISH) analyses were performed in accordance with the manufacturer’s instructions (MetaSystems)^42^. CHO mFISH probes were purchased from MetaSystems GmbH. Metaphase images were captured digitally with a CoolCubeI CCD camera and the ISIS mFISH software program (MetaSystems).

### Western blotting analysis

Western blotting was performed as described previously^43^. Membranes were blotted with rabbit monoclonal antibody against EGFP antigen (ab184601, 1:2,000, Abcam, Cambridge, UK) or with polyclonal antibody against α-tubulin (PM054–7, 1:5,000; MBL, Aichi, Japan) and the appropriate standard peroxidase-labeled anti-mouse IgG or anti-rabbit IgG secondary antibody, according to the manufacturer’s instructions (GE Healthcare). Immunoreactive bands were visualized using the ECL detection system (Pierce, Rockford, IL, USA). EGFP levels were quantified using ImageJ software.

### Genomic DNA analysis with PCR

Genomic DNA was extracted from host cells containing the HAC using a standard method^23,24^. PCR was performed using primers as follows: for CMV-Neo (318 bp), forward 5′-CGTAACAACTCCGCCCCATT-3′ and reverse 5′-GCAGCCGATTGTCTGTTGTG-3′; for internal *Gapdh* (735 bp), forward 5′-CCATCTTCCAGGAGCGAGA-3′ and reverse 5′-TGTCATACCAGGAAATGAGC-3′.

### Genomic DNA analysis with qPCR

DNA isolation and qPCR were performed as described previously^24,43^. *EGFP*, *VEGF Hc*, and *VEGF Lc* were analyzed using specific primers: for *EGFP*, forward 5′-CTTCTTCAAGTCCGCCATGC-3′, reverse 5′-GGTCTTGTAGTTGCCGTCGT-3′; for *VEGF Hc*, forward 5′-CTGCACCTGAACTTTTGGGC-3′, reverse 5′-TCGGGTGTACGGGAGATCAT-3′, for *VEGF Lc*, forward 5′-CAGTACAGTACCGTGCCCTG-3′, reverse 5′-AATACAGATGGGGCTGCGAC-3′. DNA was amplified using an Applied Biosystems StepOne thermal cycler system and a SYBR green PCR kit (Applied Biosystems, Foster City, CA, USA). Each genome sample was normalized to NV1 (NV1 CHO gPCR ref forward; 5′-ACAGGTTTCTGCTTCTGGCA-3′ NV1 CHO gPCR ref reverse; 5′-CATCAGCTGACTGGTTCACA-3′). *NV1* was previously reported as a CHO genome reference for qPCR^29^.

### ELISA

VEGF 165 antigen protein (11066-HNAH, Sino Biological, Beijing, China) was diluted with PBS to 50 ng/well, added to a 96-well plate, and incubated for 1 hr at room temperature to immobilize the antigen. After washing three times with TBS-T (Wako), 350 μl of 5% skim milk/TBS-T (Wako) was added as a blocking buffer, and plates were incubated for 1 hr at room temperature. After washing three times with TBS-T (Wako), 100 μl of each culture supernatant of CHO cells was used as a primary antibody and incubated for 1 hr at room temperature. Each clone was seeded at 2.0 × 10^5^ cells/ml in a 60-mm culture dish and cultured for 6 days (Fig. 5E,F), or each clone was seeded at 0.5 × 10^5^ cells/ml in a 60-mm culture dish and cultured for 8 days (Fig. 6B). Thereafter, the antibody was washed three times with TBS-T (Wako), and 100 μl of an antibody against human antibody (goat anti-human IgG HRP: BETHYL: A80–119 P) was diluted 1:50,000 as a secondary antibody, and allowed to stand at room temperature for 30 minutes. After washing three times with TBS-T (Wako), O-Phenylenediamine (OPD) was added to the substrate buffer as an enzyme substrate to a final concentration of 0.5 mg/ml (Thermo Fisher), and 1000 μl H_2_O_2_ (1:1000; Wako) was also added. After 30 minutes, the reaction was stopped by adding 1 M H_2_SO_4_ (Wako), and the wavelength at 492 nm was measured with an absorbance measurement plate reader (EPOCH 2, Bio Tek, Winooski, VT, USA) to numerically quantify the antibody reaction. For quantification of anti-VEGF antibody production from CHO cells, we used the concentration-defined anti-VEGF antibody, bevacizumab (Medchem Express, Monmouth Junction, NJ, USA) to create a standard curve.

### Antibody purification

Cell seed 0.5 × 10^5^ cells/ml in a 60-mm culture dish and cultured for 6 days. After the removal of cells and debris by centrifugation, the clarified culture supernatant was filtrated with 0.45 μm filter (Merck Millipore, Billerica, MA, USA). Then, 1 ml of each culture supernatant of CHO cells was used for antibody purification. Antibody purification was performed using Protein G magnetic beads (Cell Signaling Technology, Danvers, MA, USA). Added 30 μl of Protein G magnetic beads to culture supernatant and incubate with rotation overnight at 4 °C. Pellet beads using magnetic separation rack. Wash pellet three times with 1 ml of 1 × lysis buffer (Cell Signaling Technology) and one time with PBS. Resuspend the pellet with 100 μl elution buffer (0.1 M glycine, pH 2.5), briefly vortex to mix, and briefly microcentrifuge to pellet the sample. Pellet beads using magnetic separation rack. Transfer the supernatant to a new tube and the pH of the eluted fractions was adjusted to pH6.5 using 1 M Tris-HCl buffer (pH 9.0). We applied to each well the supernatant for SDS-PAGE and bevacizumab for control (Fig. 6C). WB analysis was performed using 250 ng/well of VEGF 165 protein (Sino Biological). We used 10 μl of purified antibody and bevacizumab (1:1000, Medchem Express) for first antibody and a peroxidase-labeled anti-human IgG (A80–119P, 1:4000, Bethyl Laboratories, Montgomery, TX, USA) for secondary antibody.

### Silver staining

Silver staining was performed using a Sil-Best Stain One kit (Nacalai Tesque, Kyoto, Japan) according to the manufacturer’s instructions.

### Statistics

Data from more than three separate experiments are presented as the mean ± standard deviation (SD).

## Supplementary information

Supplementary info
