# Supplementary material for: An efficient protein production system via gene amplification on a human artificial chromosome and the chromosome transfer to CHO cells
Source: Sci Rep. 2019 Nov 18;9:16954. doi: 10.1038/s41598-019-53116-2 (PMC6861226; doi:10.1038/s41598-019-53116-2)
Supplement: Supplementary file 1 — Supplementary info [file 41598_2019_53116_MOESM1_ESM.docx]

**Supplementary information**

**An efficient protein production system *via* gene amplification on a human artificial chromosome and the chromosome transfer to CHO cells**

**Takahito Ohira^1,2^, Koichi Miyauchi^1^, Narumi Uno^1,2,3^, Noriaki Shimizu^4^, Yasuhiro Kazuki^1,2^, Mitsuo Oshimura^2^, Hiroyuki Kugoh^1,2,*^**

^1^ Department of Biomedical Science, Institute of Regenerative Medicine and Biofunction, Graduate School of Medical Science, Tottori University, Yonago, Tottori, 683-8503, Japan

^2^ Chromosome Engineering Research Center, Tottori University, Yonago, Tottori, 683-8503, Japan

^3^ Department of Applied Life Sciences, Tokyo University of Pharmacy and Life Sciences, Horinouchi, Hachioji, Tokyo, 192-0392, Japan

^4^ Graduate School of Biosphere Science, Hiroshima University, Higashi-hiroshima, Hiroshima, 739-8521, Japan

* corresponding. author. Email: kugoh@med.tottori-u.ac.jp

**Supplementary Fig. S1.**


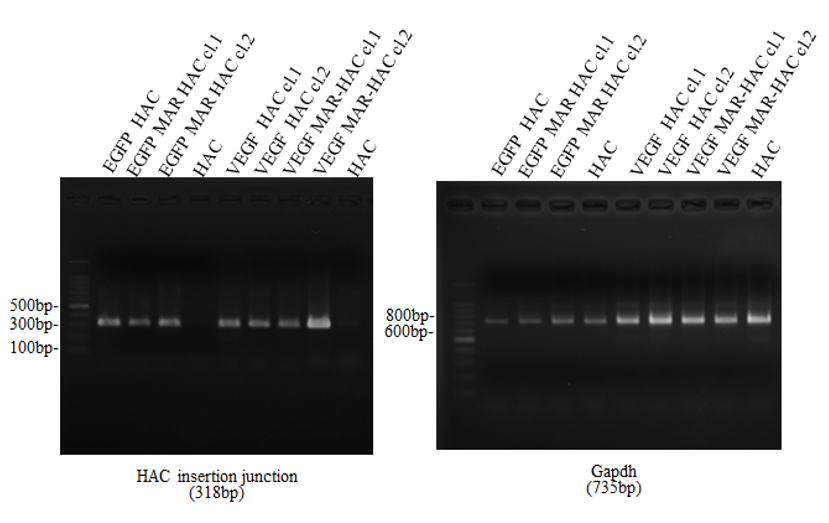


**Supplementary Fig. S1. Whole gels from Fig. 2B and Fig. 5B in the manuscript.**

The left panel is the whole gel image for PCR product of HAC insertion junction specific primers in Fig. 2B and Fig. 5B. The right panel is the whole gel image for PCR product of Gapdh specific primers in Fig. 2B and Fig. 5B.

**Supplementary Fig. S2.**


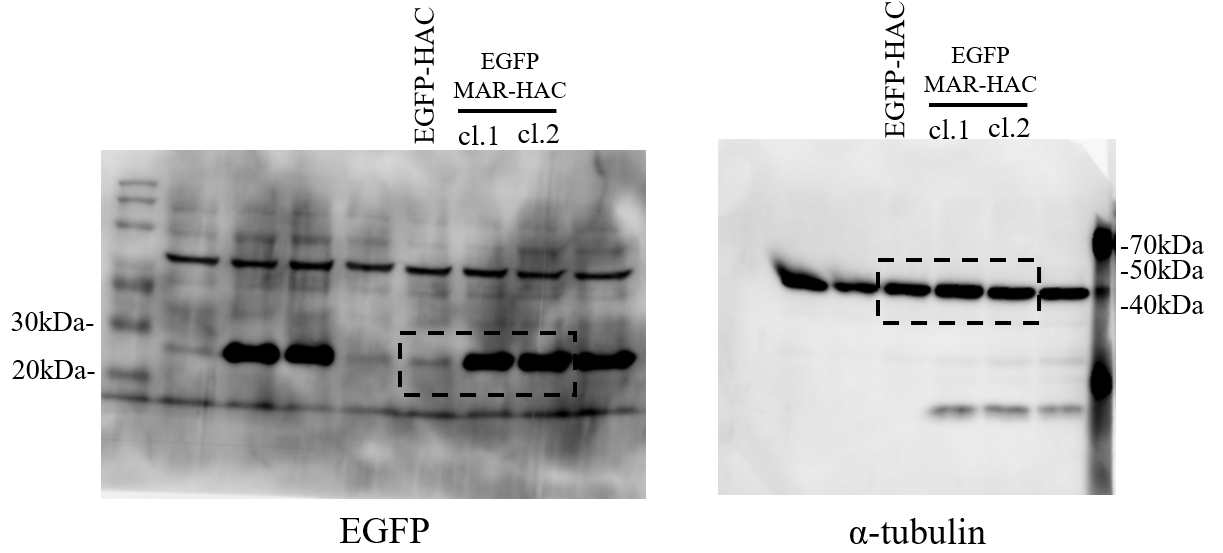


**Supplementary Fig S2. Whole western blots from Fig. 2E in the manuscript.**

The left panel is the whole gel image for western blot using EGFP antibody in Fig. 2E. The right panel is the whole gel image for western blot using α-tubulin antibody in Fig. 2E. Dotted lines show the cropped areas.

**Supplementary Fig. S3.**


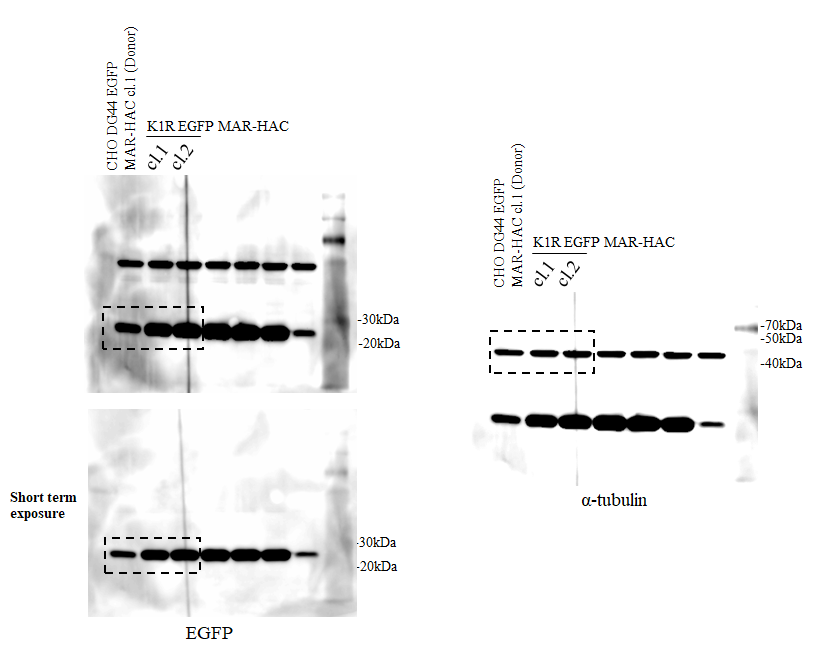


**Supplementary Fig. S3. Whole western blots from Fig. 3D in the manuscript.**

The left panel is the whole gel image for western blot using EGFP antibody in Fig. 3D. The right panel is the whole gel image for western blot using α-tubulin antibody in Fig. 3D. Dotted lines show the cropped areas.

**Supplementary Fig. S4.**


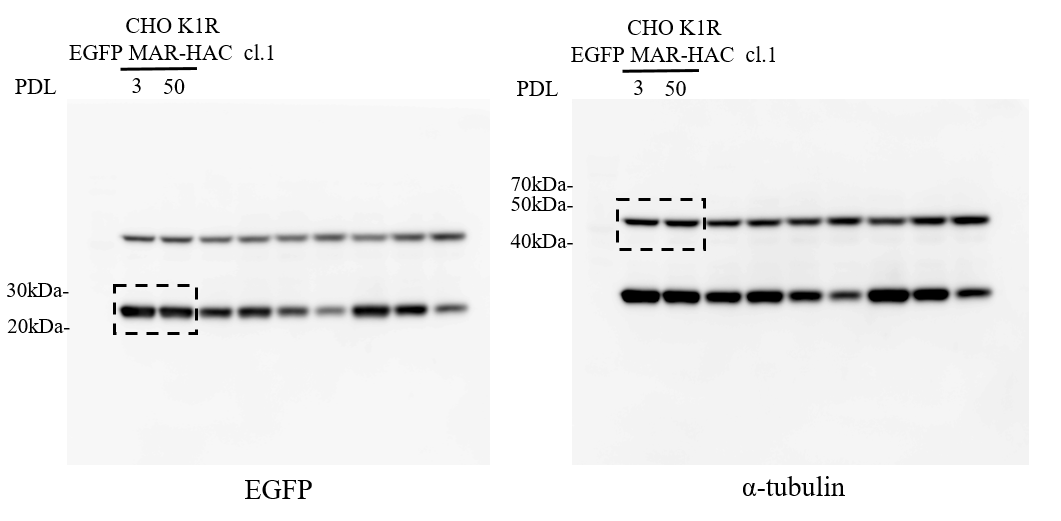

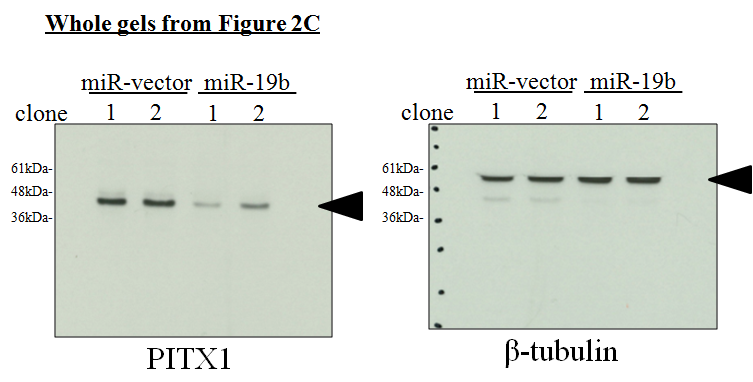


**Supplementary Fig. S4. Whole western blots from Fig. 4A in the manuscript.**

The left panel is the whole gel image for western blot using EGFP antibody in Fig. 4A. The right panel is the whole gel image for western blot using α-tubulin antibody in Fig. 4A. Dotted lines show the cropped areas.

**Supplementary Fig. S5.**


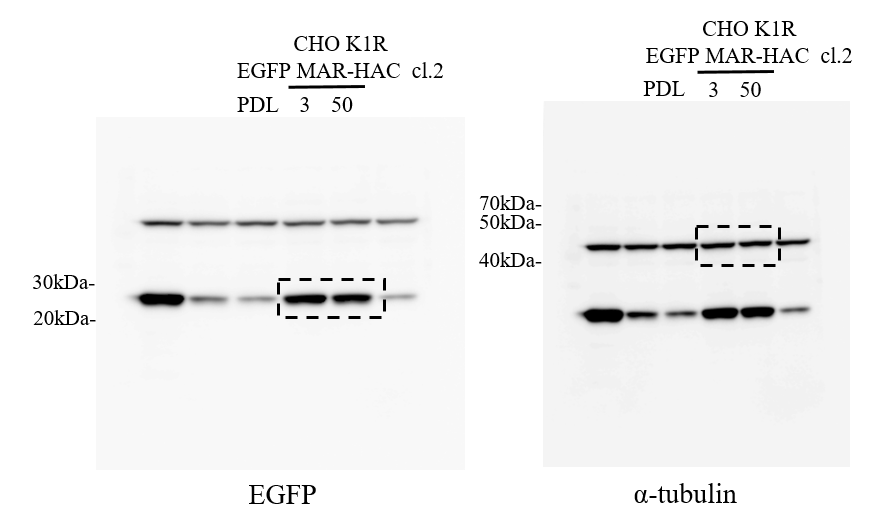


**Supplementary Fig. S5. Whole western blots from Fig. 4B in the manuscript.**

The left panel is the whole gel image for western blot using EGFP antibody in Fig. 4B. The right panel is the whole gel image for western blot using α-tubulin antibody in Fig. 4B. Dotted lines show the cropped areas.
